# Tracheobronchial Foreign Body Aspiration: Dental Prosthesis

**DOI:** 10.1155/2014/465856

**Published:** 2014-08-04

**Authors:** Ataman Köse, Dilek Kostak, Erol Aramagan, Aslıhan Durak, Nur Sezin Seçkin, Serdar Süha Dönmez, Hüseyin Melek

**Affiliations:** ^1^Department of Emergency Medicine, Faculty of Medicine, Mersin University, 33110 Mersin, Turkey; ^2^Department of Emergency Medicine, Faculty of Medicine, Uludag University, 16285 Bursa, Turkey; ^3^Department of Thoracic Surgery, Faculty of Medicine, Uludag University, 16285 Bursa, Turkey

## Abstract

It is important to extract foreign bodies for avoiding life-threatening complications. They can lead to death if they are not treated. Different signs and symptoms could occur according to the complete or partial airway obstruction. Foreign body aspiration is a rare incident in adults. The organic foreign materials such as foods are found to be aspirated more commonly and are usually settled in the right bronchial system. However, dental prosthesis and teeth aspirations are rare in literature. In our study, a 52-year-old male patient who had aspirated the front part of his lower dental prosthesis accidentally is presented and the foreign body is extracted by using rigid bronchoscopy. There are many causes of aspiration but dental prosthetic aspirations should be kept in mind during sleep. For this reason, dental apparatus must be taken out while asleep.

## 1. Introduction

Life-threatening tracheobronchial foreign body aspirations are rarely seen in adults compared to children [1]. Age groups between 6 months and 3 years old, patients who are mentally retarded and having physiological diseases, are the risky groups for foreign body aspirations. Maxillofacial trauma, unconsciousness, intoxications, dementia, using sedative drugs, and dental medications are the other risk factors for aspiration [2]. Acute respiratory distress could be seen in children but sometimes it could be asymptomatic. However, in adults, there could be productive cough, side pain, and shortness of breath as well as being asymptomatic. Sometimes this kind of asymptomatic cases could appear as a lung infection, bronchiectasis, or lung abscess in the future [1, 2]. Foods such as nuts and needles are aspirated more commonly. Teeth and dental prosthesis aspirations are rare in literature [1, 2]. It is important to retrieve the foreign body immediately and comfort the patient to avoid the complications. In children rigid bronchoscopy is commonly used but in adults both rigid and fiberoptic bronchoscopy could be used in case of aspirations [2, 3]. Here, we report the case of a 52-year-old male who aspirated his dental prosthesis during sleep and undergone successful retrieval of a dental bridge from the left main-stem bronchus using a rigid bronchoscopy.

## 2. Case Report

**Figure 1:**
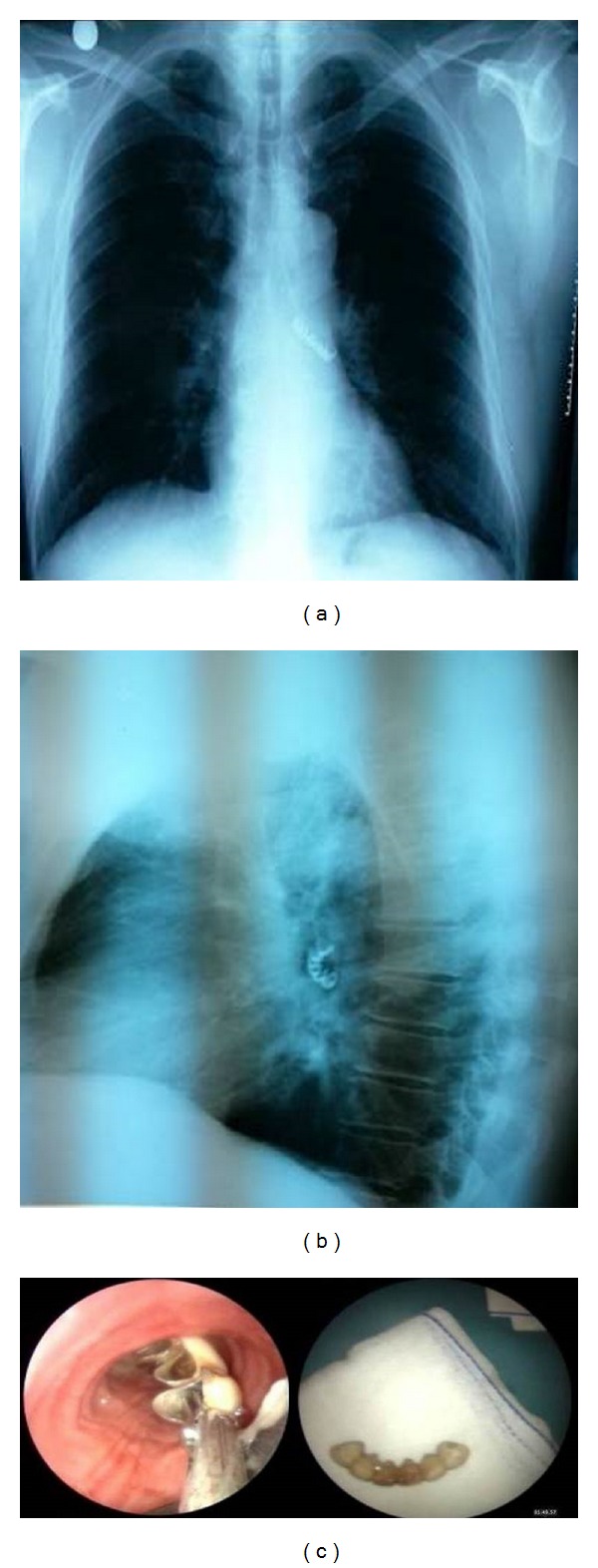
PA (a) and lateral (b) chest X-ray view in the left main bronchus radiopaque foreign body was considered as a dental prosthesis. (c) Extracted dental prosthesis with bronchoscopy.

A 52-year-old male patient was referred to the emergency department having cough attacks while sleeping. He was stable, he had not any active complaint, and his saturation was 96% with pulse oximetry. He confirmed not getting any sedative drugs and aspiration happened while he was asleep. He was taking antihypertensive drug therapy and in physical examination he was conscious, cooperating, and oriented. In auscultation the breathing sounds were low in the left lung. The laboratory tests were within the normal limits. The foreign body as a dental prosthesis was seen in the left main-stem bronchus in the posterior-anterior (PA) lung graph and lateral side lung graph (Figures 1(a) and 1(b)). The patient was referred to the thoracic surgery department and immediate bronchoscopy was performed. Trachea and left main bronchus were entered using 8.5 rigid bronchoscopy, under general anesthesia, and the foreign body was retrieved by using coaxial and optic bronchoscopy (Figure 1(c)). Following this, the secretions were cleaned and the procedure was ended by confirming there was no bleeding. The patient was extubated and admitted to the intensive care unit. *β*-Adrenergic agonist drugs and oxygen were given in case of having respiratory distress. After 7 hours of observation, the control lung graph was taken and he was discharged from hospital without any problem.

## 3. Discussion

Tracheobronchial foreign body aspirations are serious medical problems leading to death. Although they can be seen in every age group, they are less common in adults compared to children [4]. The risk factors are mental retardation, maxillofacial trauma, unconsciousness, intoxications, dementia, using sedative drugs, and dental prosthesis. However, our report of aspiration of a fixed dental prosthesis differs from the published case reports as being not related to any of the mentioned causes above. This case of dental prosthesis aspiration had occurred in an otherwise healthy adult during his sleep. Therefore, we consider that this original case report may shed some light on at least some of the undefined aspirated foreign bodies in lungs that are discovered on chest radiographs.

Anamnesis is important in case of tracheobronchial aspiration. Coughing is the most frequent complaint. In the beginning, it is nonproductive, irritative, and spasmodic and comes as cough attacks. Later with the implantation of the foreign body into the bronchus, severity and characteristics are resolved [5]. 59–82.5% of the patients have this kind of coughing [4, 6]. Our patient presented with cough which is the most common presenting symptom. Foreign body aspiration should always be kept in mind with the patients having symptoms such as respiratory distress, chronic cough, voice problems, recurrent lung infections, atelectasis, chronic lung infections, abscess, and complications like bronchiectasis [1, 3]. However, such complications had not happened in our patient.

Radiological assessments should be performed if there is suspicion of aspiration. First of all, PA and lateral lung graph should be taken after getting anamnesis and physical examination if there is suspicion of tracheobronchial foreign body aspiration [1, 7]. Radioopaque materials could be seen directly but radiolusense ones could not be seen. Also, pneumothorax and pneumomediastinum should be kept in mind in suspected cases [7, 8]. If the aspirated material is radioopaque it could be seen directly in posteroanterior lung graph. However, in case of getting the exact size and location of the foreign body, lateral and oblique graphs should be used. Normal views in posteroanterior lung graphs have been reported in 25% of the cases [8]. In a review of 59 patients, Limper and Prakash [1] found that routine chest radiographs are successful in locating aspirated foreign bodies in 41 of 57 cases.

The location of the foreign body is related to the structure of tracheobronchial tree and the patient's posture [3]. Right main and distal bronchus are the most frequent parts [9]. Tracheobronchial foreign body aspirations are much more frequent to the right side because of being shorter than left bronchus, wider than left side, and more vertical like trachea. In a study presented by Pasaoglu et al., it was reported that foreign body aspirations are 49.4% in right part of the tracheobronchial tree especially in right main bronchus [6]. Debeljak et al. reported that 67% of foreign body aspirations are to the right bronchus and 32% are to the left bronchus [10]. Carluccio and Romeo have reported that lower lobe of the right lung is the most frequent aspiration place followed by lower lobe of the left lung [11]. In the case reported by Başoglu and friends, the aspirated dental prosthesis is placed in the right main bronchus [12]. However, as in this case report, foreign bodies may enter the left main-stem bronchus and could have been reported in all airway locations.

Bronchial foreign bodies vary according to their natures. Bones, foods, metallic objects, dental prosthesis, teeth, and plastic objects are the most frequent ones [10, 13]. In the study carried out by Limper and Prakash, dental objects are found to be the second most frequent cause of aspiration [1]. Dental aspirations could occur during dental procedures and due to ethanol intoxication, maxillofacial trauma, stroke, dementia, decreased gag reflex, and Parkinson's disease in elderly population. Other risk factors are local anesthetic and iv sedative drug use [12, 13].

Bronchoscopy has a great value in retrieving the foreign bodies. Rigid and fiberoptic ones could be used in this kind of situations [9–13]. 97% of the foreign bodies have been reported to be retrieved by bronchoscopy. In some kind of cases surgical procedures could be needed [10]. Rigid bronchoscopy is done under general anesthesia and airway control is held safely [1, 12]. Fiberoptic bronchoscopy in retrieving the foreign bodies from tracheobronchial tree is not an accepted approach [6]. Limper and Prakash have reported that fiberoptic bronchoscopy is successful in 60% of the cases although rigid bronchoscopy is successful in 98% of cases as fiberoptic bronchoscopy has a good view and manipulation. Fiberoptic bronchoscopy should be used in some kind of specific situations like retrieving foreign bodies in peripheric bronchus and in patients with cervicofacial and maxillofacial traumas where the cervical movements are restricted [1, 13]. In our case, rigid bronchoscopy is used.

In conclusion, the most frequent complaint related to the tracheobronchial foreign body aspiration is coughing. After it is implanted in the bronchus the characteristic and the severity of the cough dissolve. In this kind of cases aspiration should always be kept in mind. There are many reasons of aspirations but dental prosthetic aspirations should be remembered if it happened while sleeping. For his reason, dental apparatus must be taken out while asleep. The place of aspiration is related to the structure of the tracheobronchial tree and the patient's posture during aspiration. The foreign body could be placed in the left main bronchus. Posteroanterior and lateral lung graphs are the first choices if there is suspicion, but for the exact diagnosis, bronchoscopy should be done. History, physical examination, and radiological assessments are generally adequate for the suspicion of foreign body aspiration. However, in the patients having lung problems with no clinical signs and medical history, if there is a suspicion of aspiration, bronchoscopy is indicated.
